# Functional Identification of *EjGIF1* in *Arabidopsis* and Preliminary Analysis of Its Regulatory Mechanisms in the Formation of Triploid Loquat Leaf Heterosis

**DOI:** 10.3389/fpls.2020.612055

**Published:** 2021-01-12

**Authors:** Chao Liu, Renwei Huang, Lingli Wang, Guolu Liang

**Affiliations:** ^1^ College of Basic Medical Sciences, Chengdu University of Traditional Chinese Medicine, Chengdu, China; ^2^ Sichuan Provincial Key Laboratory for Development and Utilization of Characteristic Horticultural Biological Resources, College of Chemistry and Life Sciences, Chengdu Normal University, Chengdu, China; ^3^ Technical Advice Station of Economic Crop, Chongqing, China; ^4^ College of Horticulture and Landscape Architecture, Southwest University, Chongqing, China

**Keywords:** triploid loquat, EjGIF1, transgenic Arabidopsis, leaf size, heterosis, DNA demethylation

## Abstract

Although several results have been obtained in triploid loquat heterosis (i.e., leaf size of triploid loquat) studies in the past years, the underlying mechanisms of the heterosis are still largely unknown, especially the regulation effects of one specific gene on the corresponding morphology heterosis. In this study, we sought to further illustrate the regulatory mechanisms of one specific gene on the leaf size heterosis of triploid loquats. A leaf size development-related gene (*EjGIF1*) and its promoter were successfully cloned. Ectopic expression of *EjGIF1* in *Arabidopsis* showed that the leaf size of transgenic plantlets was larger than that of WTs, and the transgenic plantlets had more leaves than WTs. Quantitative Reverse Transcription PCR (qRT-PCR) showed that the expression level of *EjGIF1* showed an AHP expression pattern in most of the hybrids, and this was consistent with our previous phenotype observations. Structure analysis of *EjGIF1* promoter showed that there were significantly more light-responsive elements than other elements. To further ascertain the regulatory mechanisms of *EjGIF1* on triploid loquat heterosis, the methylation levels of *EjGIF1* promoter in different ploidy loquats were analyzed by using bisulfite sequencing. Surprisingly, the total methylation levels of *EjGIF1* promoter in triploid showed a decreasing trend compared with the mid-parent value (MPV), and this was also consistent with the qRT-PCR results of *EjGIF1*. Taken together, our results suggested that *EjGIF1* played an important role in promoting leaf size development of loquat, and demethylation of *EjGIF1* promoter in triploid loquats caused *EjGIF1* to exhibit over-dominance expression pattern and then further to promote leaf heterosis formation. In conclusion, *EjGIF1* played an important role in the formation of triploid loquat leaf size heterosis.

## Introduction

Heterosis, or hybrid vigor, is a common phenomenon in many diploid or polyploid organisms, which means the biomass, resistance ability, yield, and some other agronomic traits in hybrids are greater than that of the parents (Hofmann, 2012). Heterosis has been widely used to improve the yield of the field crops and vegetables continuously and thus has greatly solved the crisis of food shortage especially in some developing countries (Agbo and Teixeira da Silva, 2014). However, to date we still know little about the mechanisms of heterosis (Wang et al., 2015). Researchers have proposed several models from the genetic aspect to explain the mechanisms of heterosis including dominance, over-dominance, and epistasis, but none of these models can fully explain this phenomenon (Jones, 1917; East, 1936; Yu et al., 1997). Recent studies on maize, soybean, rice, *Arabidopsis*, etc., have found that heterosis may be associated with the differential gene expression based on the fact that no new genes are produced after hybridization (Guo and Rafalski, 2013; Miller et al., 2015; Wang et al., 2015; Taliercio et al., 2017; Chen et al., 2018). Two gene expression-related models, additive and non-additive gene expression, were proposed by Chen (2010) to further explain heterosis phenomenon. With the development of functional genomics, such as the application of RNA-Seq technology, more and more studies have found that heterosis may be highly related to additive expression pattern due to the fact that genes exhibit non-additive expression pattern in hybrids are comparatively rare, and the non-additive genes are deemed to associate with the formation of transgressive traits in hybrids (Guo et al., 2006; Thiemann et al., 2014). For instance, study on triploid loquat, Liu et al. (2018a) analyzed the leaf transcriptomes of the triploid loquats and their parents in two cross combinations and identified that 94.56 and 86.97% transcripts were expressed additively in the two cross combinations, respectively, and only 5.44 and 13.03% genes expressed non-additively. These results indicated that additively expressed genes may play a fundamental role in the formation of triploid loquats.

Recent studies found that epigenetic mechanisms, especially DNA methylation which are considered to be associated with the regulation of gene expression in a number of plant species (Arikan et al., 2018). Due to the regulatory function on gene expression, DNA methylation level is also considered to be associated tightly with heterosis (Nakamura and Hosaka, 2010). Studies have shown that DNA methylation is mainly occurred in the CpG island of the promoter, and the DNA methylation density of a promoter can affect the transcriptional activity of the gene (De Smet et al., 1999; Alasaari et al., 2012).

Loquat [*Eriobotrya japonica* (Thunb.) L.; 2n = 2x = 34] belongs to the subtribe Pyrinae in the Rosaceae family and is favored by many people due to its excellent flavor and medicinal applications (Wu et al., 2015). However, the loquat fruits sold in the market currently are all diploid with too many seeds, and this significantly affects their edibility (Liu et al., 2018a). Triploid loquat breeding provides a new way to solve the problem of low edible rate of diploid loquats. Previous studies in our lab found that triploid loquats are not only seedless, but also have a variety of excellent traits that diploid and tetraploid loquats do not have, such as larger and greener loquat leaves, showing an obvious heterosis (Liu et al., 2018b, 2019). Liu et al. (2018a,b) have studied the mechanisms of triploid loquat heterosis by using several triploid loquats with clear genetic relationship and found that extensive genetic variation and DNA methylation remodeling after the formation of triploid loquat may change the gene expression patterns in triploid loquats, and these further promoted the formation of triploid loquat heterosis. However, for triploid loquat heterosis, we still know little about the mechanisms.

Leaves are the photosynthetic place of plants, absorbing sunlight energy to synthesize biological energy (Gonzalez et al., 2012; Jiao et al., 2019). Leaves of eudicots are initiated at the flank of the shoot apical meristem (SAM), and the extent and direction of leaf growth have a great influence on the leaf size and shape (Horiguchi et al., 2005; Vercruyssen et al., 2015). Plant Growth-Regulating Factor (GRFs) is a family of transcription factors that regulate leaf development, and nine GRFs (GRF1-GRF9) were identified from *Arabidopsis* (Kim et al., 2003). Studies on *Arabidopsis* and rice found that GRFs could repress or activate the expression of their target genes by binding to the regulatory region of DNA (Kim et al., 2012; Kuijt et al., 2014). Overexpression of *AtGRF1*, *AtGRF2*, and *AtGRF5* could lead the cell number or size of transgenic leaves to decrease, and these make the transgenic plants have larger leaves than wild-type (WT) plants (Kim et al., 2003). GRF INTERACTING FACTOR 1/ANGUSTIFOLIA 3 (GIF1/AN3) is a transcriptional coactivator which is a functional homolog to the human synovial sarcoma translocation protein (SYT) transcription coactivator (Horiguchi et al., 2005; Vercruyssen et al., 2015). Overexpression of *AtGIF1* enlarged the leaf size of the transgenic plants, whereas, loss-of-function *gif1* plants developed narrower leaves (Kim and Kende, 2004; Horiguchi et al., 2005). Yeast two-hybrid analysis showed that AtGIF1 could interact with both AtGRF1 and AtGRF5, and positively promoted the leaf cell proliferation and regulated the leaf size in plants (Kim and Kende, 2004; Horiguchi et al., 2005). Thus, like GRFs, GIF1 also functions as an important transcription factor in the size and shape regulation of plant leaves (Kim and Kende, 2004).

Although we have verified that the triploid loquat leaves become larger, greener than that of diploid and tetraploid loquats, showing an obvious heterosis (Supplementary Material: Supplementary Table S1), we still know little about the association of leaf development with triploid loquat leaf heterosis and also few reports on this issue. Illuminating the mechanisms of leaf development of loquat could help us better understand the heterosis phenomenon of triploid loquat leaf and provide more details for the triploid loquat application in loquat breeding. In this study, we have identified the transcription factor EjGIF1 in loquat and made a further validation for *EjGIF1* function, and at the same time, *EjGIF1* promoter was cloned and also the methylation level of *EjGIF1* promoter was analyzed by bisulfite sequencing (BSP) in different ploidy loquats. Our study will provide more information on the morphology heterosis of triploid loquat leaf.

## Materials and Methods

### Plant Lines

In order to overcome the unclear origin of loquat, the triploid loquats used in this study were created by cross-fertilizing. Two triploid loquat lines were generated in 2003, named Triploid-A and Triploid-B. For the two triploid lines, the same female parent (Longquan-1 tetraploid) was used to cross with two different wild diploid loquats, GC-1 (Triploid-A) and GC-23 (Triploid-B). The tetraploid parent Longquan-1 was selected by our laboratory, while the wild diploid parents, GC-1 and GC-23 were identified in Guizhou Province, China, which grow naturally in the rocky arid region and have strong levels of abiotic and biotic resistance (Wu et al., 2015). In the meantime, GC-1 and GC-23 also have a far genetic distance with cultivated loquats, which could increase mutations in triploid loquats after hybridization (Wu et al., 2015). Finally, nine and three triploid loquats were obtained in Triploid-A and Triploid-B, respectively, which were labeled as A-1, A-2, A-3,… A-9 and B-1, B-2, B-3. All the plants were grown in a natural environment, in the Experimental Base of College of Horticulture and Landscape Architecture, Southwest University, Chongqing, China.

### Isolation of *EjGIF1* Complementary DNA Sequence

The reference sequence of *EjGIF1* was obtained from the RNA-Seq data base in our laboratory. The leaf material of Longquan-1 tetraploid was used for the cDNA isolation; moreover, the RNA extraction and cDNA synthesis methods were performed the same as Liu et al. (2019). The cloning primers (*EjGIF1*-*Asc* I-F and *EjGIF1*-*Xba* I-R) were designed based on the *EjGIF1* reference sequence, and the restriction enzyme sites were added at the 5'-end and 3'-end for the subsequent vector construction (Table 1). PCR products were then cloned to the pMD19-T (Takara, Dalina) for sequencing.

**Table 1:** Primers used in this study.

Primer name	Primer sequence (5'-3')	Tm value (°C)
*EjGIF1*-*Asc* I-F	5'-AGGCGCGCCATGCAGCAGCACCTGATCAGA-3'	87.3
*EjGIF1*-*Xba* I-R	5'-GCTCTAGATTAATTTCCATCATCGGTCGAT-3'	68.6
1-*EjGIF1* GSP1	5'-TGGTAGGAGGCTGGGGTTGAGAATC-3'	68.7
1-*EjGIF1* GSP2	5'-GCTGTAGCTTTGCTTGGTTCTCTGC-3'	66.0
2-*EjGIF1* GSP1	5'-CTCTCTCTAACTTTCTCACTCC-3'	49.6
2-*EjGIF1* GSP2	5'-GCTTTTTTTTTACAGAGTTGAG-3'	51.9
3-*EjGIF1* GSP1	5'-TTGCTGCATGTAATGTGCTCCTGGTTG-3'	71.1
3-*EjGIF1* GSP2	5'-AGATTCCGCTGTAGCTTTGCTTGGTTC-3'	69.4
4-*EjGIF1* GSP1	5'-AAGAAGGAGGACCTGCTGAATGTGATC-3'	67.4
4-*EjGIF1* GSP2	5'-GTTGTTAGGATAATAGGCTGCCATCAT-3'	63.8
5-*EjGIF1* GSP1	5'-CAGATTGTTGAGATGTTTATTGCGGGC-3'	69.1
5-*EjGIF1* GSP2	5'-AATGGCGTACAGAGAATGCGATTGTCA-3'	69.9
q*EjGIF1*-F	5'-TACTCCCAGCAACCGTTTTCA-3'	60.7
q*EjGIF1*-R	5′-TCCAGCATTATTTCCCTCATT-3'	56.7
*EjGIF1*-F	5'-ATGCAGCAGCACCTGATG-3'	55.1
*EjGIF1*-R	5'-TTAATTTCCATCATCGGTCGAT-3'	51.5
CaMV 35s_F	5'-TGAGACTTTTCAACAAAGGATAATT-3'	54.6
CaMV 35s_R	5'-TGTCCTCTCCAAATGAAATGAAC-3'	58.5
CpG1-F	5'-ACAGTTACCTGAGGACTCTGGAGTC-3'	64.4
CpG1-R	5'-CTGTGGTAGTGAGAAGTAAGGTCGT-3'	65.2
CpG2-F	5'-CCACAGTAAGTACAACCACCAG-3'	59.6
CpG2-R	5'-CTCAAACAGATCGTGTCTACACTTT-3'	58.5
CpG3-F	5'-GGTTTTGTAGGTAAGATTATAGATTTGAGA-3'	61.6
CpG3-R	5'-TAAAAATAATCCCCAACCACCTATA-3'	59.4

### Isolation and Analysis of *EjGIF1* Promoter Sequence

In order to analyze the structure and methylation level of *EjGIF1 Cis*-element, the promoter sequence of *EjGIF1* was isolated based on the user manual of Universal Genome-Walker Kit 2.0 (Takara, Clontech Laboratories, Inc., Japan). The nested primers (1-*EjGIF1* GSP1 to 5-*EjGIF1* GSP2) used for promoter cloning were listed in Table 1, and the amplification products were sequenced as the same as described above. The possible regulatory elements of the *EjGIF1* promoter were annotated by using the PlantCARE database.

### Expression Pattern Analysis of Loquat *EjGIF1* Gene in Different Ploidy and Developmental Stages

To analyze the expression level of *EjGIF1* in different ploidy loquats, and in different developmental stages of loquat leaves as well, leaves from three developmental periods of different ploidy loquats were collected and named P I (young leaves < 5 cm), P II (5 cm < medium mature leaves < 15 cm) P III (mature leaves), respectively (Gong et al., 2014). The expression levels in different developmental stages were analyzed by using the materials of P I, P II, and P III. The RNAs were extracted as described by Liu et al. (2019). cDNA synthesis and Quantitative Reverse Transcription-PCR (qRT-PCR) methods were also performed by using the methods as described by Liu et al. (2019). The primers (q*EjGIF1*-F and q*EjGIF1*-R) used in qRT-PCR analysis were listed in Table 1. *Actin* of loquat was analyzed with the primer sequences 5'-ATCCTTCGTCTGGACCTTGC-3' and 5'-GACAATTTCCCGTTCAGCAGT-3'. All of the samples were examined in triplicate.

### *EjGIF1* Overexpression Plasmid Construction and *Arabidopsis* Transformation

The full length cDNA sequence of *EjGIF1* was cloned to *Asc* I-*Xba* I sites of pFGC5941 plasmid so that the *EjGIF1* could express under the control of CaMV 35S promoter. The recombinant plasmid pFGC5941-35S::*EjGIF1* was then transferred to the *Agrobacterium tumefaciens* strain LBA4404 by means of electric shock. Afterward, WT plants were transformed by using the floral dip method (Clough and Bent, 1998) with minor modifications. Infiltration media used contained 5% sucrose and 0.02% Silwet. Seeds of transgenic lines (T0) were planted in soil and were selected by spraying with 20 g/L glufosinate-ammonium after 2 weeks. The same selection methods were used until the T2 generation was obtained, and the T2 homozygous progenies were used for phenotype observation and expression test of *EjGIF1* by qRT-PCR. All the seedlings with glufosinate-ammonium resistance were grown in a growth chamber under the 16 h light/8 h dark photoperiod (2,500 lux).

### Positive Transgenic Plantlet Verification

Genomic DNAs were isolated from young, fresh leaves of glufosinate-ammonium resistance plants and WT plants with a modified cetyltrimethyl ammonium bromide (CTAB) method (Liu et al., 2005). Then, PCR was carried out for detecting the insertion, and the WT was used as a control. The transgenic and WT plants were tested for the presence of both *EjGIF1* and CaMV 35s genes separately, and primers (*EjGIF1*-F and *EjGIF1*-R, and CaMV 35s_F and CaMV 35s_R) are listed in Table 1.

### Gene Expression Detection in the Positive and Wild Type Plants

Total RNAs were extracted from young, fresh leaves of T2 homozygous progenies and WT plants. The RNA extraction, cDNA synthesis and qRT-PCR methods were performed the same as Liu et al. (2019). The primers (q*EjGIF1*-F and q*EjGIF1*-R) were listed in Table 1. WTs were used as controls and the reference gene (*Actin*) of *Arabidopsis* was analyzed with the primer sequences 5'-CTTCGTCTTCCACTTCAG-3' and 5'-ATCATACCAGTCTCAACAC-3'. Each transgenic line and each WT was examined in three plantlets as biological repetition.

### Leaf Morphology Traits Analysis of Transgenic and WT Plants

The T2 homozygous progenies and WT plants were grown in the growth chamber for about 1 month and their leaf morphology traits were recorded individually. The methods for leaf length and width measuring were the same as Liu et al. (2018b), and the leaf size was measured by using the ImageJ software. For each trait, 20 individuals in each transgenic line were measured as biological repetition, and three values were measured for each individual as technical repetition.

### BSP Sequencing for *EjGIF1* Promoter in Different Ploidy Loquats

Tiangen Bisulfite Conversion Kit (Tiangen Company, Beijing) was adopted for genomic DNA bisulfite conversion. CpG islands prediction and PCR amplification primers for bisulfite sequencing design were carried out by using the online software (http://www.urogene.org/cgi-bin/methprimer/methprimer.cgi). Primers (CpG1-F and CpG1-R, CpG2-F and CpG2-R, and CpG3-F and CpG3-R) used for bisulfite sequencing are listed in Table 1. The amplification products were also sequenced as the same as described above, and for each CpG island, 15 randomly chosen clones per genotype were sequenced. The methylation levels were counted as described by Liu et al. (2018b).

### Statistical Analysis

The phylogenetic tree was generated by using the Clustal W, and the bootstrap test was set at 1,000 to test confidence for the tree (Higgins et al., 1994). The MEGA 5.0 software was used for phylogenetic tree construction with Neighbor-Joining method (Tamura et al., 2011). Mid-parent value (MPV) was adopted to measure the heterosis, and it was calculated by using the method of Turner (1953). Briefly, MPV was calculated according to the genomic contribution by the two parents, i.e., MPV = 2/3 Longquan-1tetraploid + 1/3 GC-1/GC-23. The gene expression patterns were classified into two classes by using the method described by Liu et al. (2019). Briefly, (i) additive expression pattern, which gene expression levels in hybrids were at the MPV (MPL); (ii) non-additive expression pattern, which gene expression level was deviated from the MPV. The non-additive expression pattern was further classified into two classes; (iii) dominance expression pattern, which the gene expression level was at the high parent level (HPL) or at the low parent level (LPL); and (iv) over-dominance expression pattern, which the gene expression level was above the high parent level (AHP) or below the low parent level (BLP). Finally, the significance examination was performed by using the one-way ANOVA method.

## Results

### Identification and Characterization of *EjGIF1*

**Figure 1:**
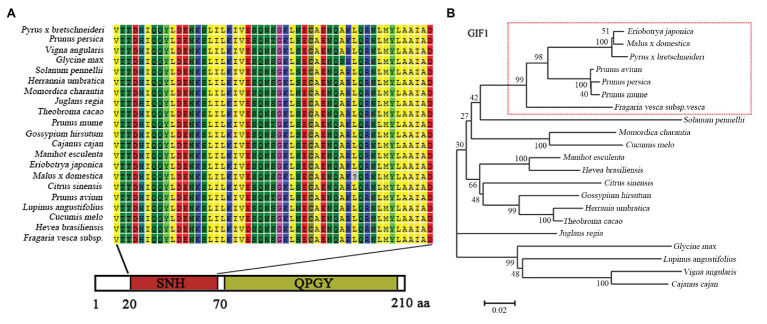
**(A)** Multiple sequence alignment of EjGIF1 SNH domains with other GIF1s reported in National Center for Biotechnology Information (NCBI); **(B)** Phylogenetic relationships between loquat EjGIF1 and other GIF1 proteins reported in NCBI. The unrooted were constructed using MEGA 5.0 by neighbor-joining method, and a bootstrap test was set at 1,000 to test confidence for the tree.

Based on the reference sequence from RNA-Seq database, a segment of 651 bp cDNA sequence was obtained and sequenced, named *EjGIF1*. Sequence analysis showed that *EjGIF1* encoded a 216 amino acids protein with the molecular weight of 23.20 kDa. The sequence of *EjGIF1* was submitted to National Center for Biotechnology Information (NCBI) and the accession number was MK573556. To investigate the relationship among the *GIF1* genes in different species, we downloaded the reported cDNA sequences from NCBI, and these reported GIF1 proteins were mainly distributed in 10 families, Rosaceae, Solanaceae, Curcurbitaceae, Euphorbiaceae, Malvaceae, Sterculiaceae, Leguminosae, Rutaceae, Juglandaceae, and Papilionoideae. The phylogenetic tree was created by using the deduced protein of EjGIF1 and these reported GIF1 proteins. The same with the traditional taxonomy, our results showed that EjGIF1 was separated from the other GIF1 proteins clearly, and EjGIF1 was clustered into Rosaceae and was closest to *Malus* × *domestica* (Figure 1B).

GIF1 is a leaf shape related protein which was first isolated by Relichova (1976). Previous researches showed that GIF1 is a homolog of SYT whose N-terminal contains a conserved SYT N-terminal homology (SNH) domain, and this domain could participate in protein-protein interactions (Crew et al., 1995; Thaete et al., 1999; Kato et al., 2002). In this study, results of multiple sequence alignment showed that EjGIF1 also contained a SNH domain, and this was consistent with the previous studies (Figure 1A). Taken together, these results suggested that EjGIF1 gene is kept highly conserved during the evolution processes.

### Generating and Verification of Transformants

To investigate the potential function of *EjGIF1*, an over-expression vector with *EjGIF1* CDS sequence under the control of CaMV 35S promoter was transferred into *Arabidopsis*. After continuous screening with glufosinate-ammonium, we finally got 50 plantlets belonging to 10 transgenic lines. The transgenic seedlings and the WT ones were then transferred to the new pots and cultured in the growth chamber.

**Figure 2:**
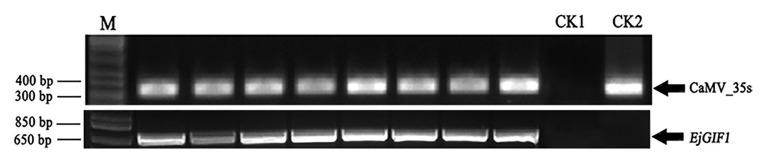
Positive transgenic plantlets verification. CaMV_35s and *EjGIF1* genes were detected by PCR, and CK1 and CK2 were set as control. M represents Marker; CK1: genome DNA of wild type; CK2: empty pFGC5941 plasmid.

To verify the reliability of the transgenic plantlets, the presence of *EjGIF1* and CaMV_35s in the genomes of transgenic and WT plantlets were performed by PCR separately. The empty vector (pFGC5941) and the WT genomic DNA were set as controls. The detection results of the two genes in the transformants and WTs suggested that the two expected specific fragments appeared in the right positions, indicating the precision of these transgenic plantlets was reliable (Figure 2).

### Over-Expression of *EjGIF1* in *Arabidopsis* Enlarged Leaf Size and Leaf Number

**Figure 3:**
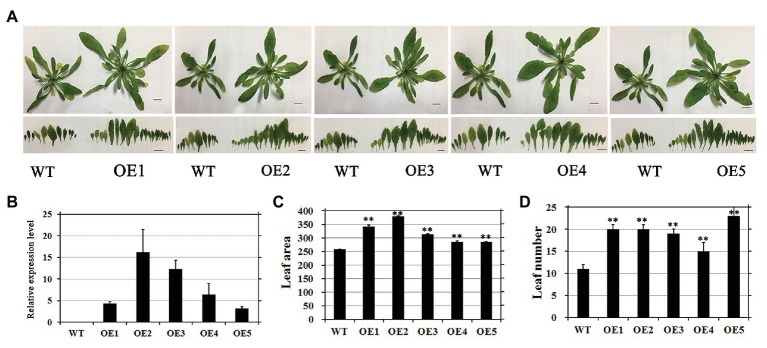
**(A)** Phenotypes of transformants and WT plants. The size of the bar showed in the picture was 1 cm. **(B)** Expression analysis of *EjGIF1* in T2 homozygous progenies and WTs. *Actin* gene was selected by our laboratory previously which was used as a control. All data are from three biological repeats (*n* = 3). **(C)** Leaf area analysis of the transgenic plantlets and WTs. **(D)** Leaf number analysis of the transgenic plantlets and WTs. ^**^Represents the significance level of the one-way ANOVA test, *p* = 0.01. Error bars denote |S|D.

To evaluate the regulatory effects of *EjGIF1* on leaf development in *Arabidopsis*, we selected five independent transgenic lines (named: OE1, OE2…OE5) with fine phonetype for further phenotype analysis, and the WT plantlets were set as controls. The transgenic and WT plantlets were grown in a growth chamber for about 1 month. The same to the previous studies, we also found that overexpression *EjGIF1* in *Arabidopsis* could lead the leaf length and width to become larger than the WTs (Table 2). Moreover, the leaf sizes of the transgenic plantlets were enlarged as well. As shown in Table 2, the leaf area of the five transgenic plantlets (OE1, OE2…OE5) were 342.53 mm^2^, 380.32 mm^2^, 313.00 mm^2^, 285.72 mm^2^, and 285.48 mm^2^, while the WT was 257.71 mm^2^ (Figures 3A,C; Table 2). Correlation analysis between the leaf area and the expression level of *EjGIF1* in the five transgenic plantlets found that except for OE1, there was a positive correlation between the leaf area and the expression level of *EjGIF1* in the transgenic plantlets (Figures 3B,C). Therefore, these indicated that *EjGIF1* plays an important role in regulating the development of the loquat leaf size.

**Table 2:** Leaf morphologies analysis of the transgenic and WT plantlets.

	WT	OE1	OE2	OE3	OE4	OE5
Leaf length (cm)	3.3 ± 0.1^a^	4.3 ± 0.1	4.5 ± 0.1	4.3 ± 0.1	4.0 ± 0.1	4.7 ± 0.1
Leaf width (cm)	1.2 ± 0.1	1.3 ± 0.1	1.6 ± 0.1	1.4 ± 0.1	1.4 ± 0.1	1.3 ± 0.1
leaf area (mm^2^)	257.71 ± 1.36	342.53 ± 4.20	380.32 ± 0.91	313.00 ± 1.55	285.72 ± 3.32	285.48 ± 1.01
Leaf number	11 ± 1	20 ± 1	20 ± 1	19 ± 1	15 ± 2	23 ± 2

a mean ± standard deviation.

Interestingly, we also found that the transgenic plantlets had significantly more leaves than the WT (Figure 3D; Table 2). As shown in Table 2, the WT contains 11 leaves, while the transgenic plantlets contain 20, 20, 19, 15, and 23 leaves, respectively. Different from previous studies (Kim and Kende, 2004; Horiguchi et al., 2005), our results suggested that *EjGIF1* could not only promote the development of leaf size but also increase the formation of leaf primordium, but how does this occur requires to be further researched.

Finally, the expression levels of *EjGIF1* in the five transgenic lines and WTs were detected by qRT-PCR. Results showed that *EjGIF1* were expressed higher in all the five transgenic lines than that of the WTs (Figure 3B), and transcripts have not been detected out in the WT ones.

### Expression Analysis of *EjGIF1* in Different Developmental Stages and Ploidy Loquat

**Figure 4:**
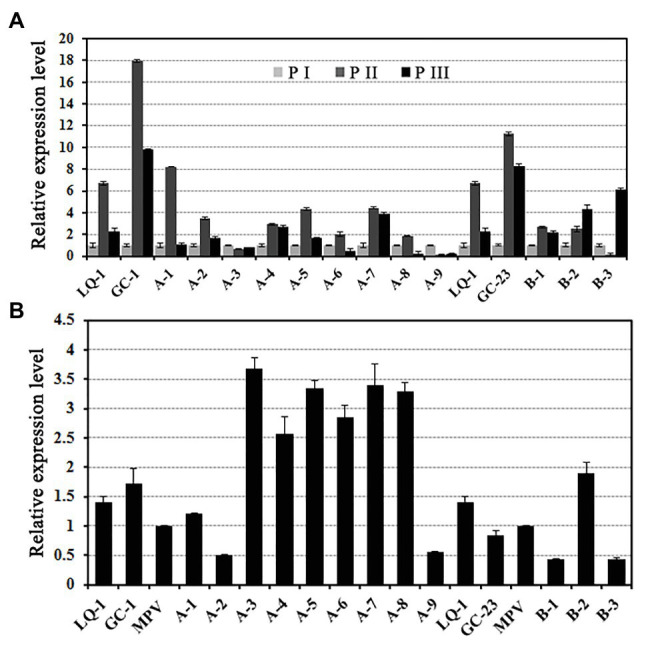
**(A)** *EjGIF1* expression analyses in different development stages and **(B)** ploidy loquats. *Actin* gene was selected by our laboratory previously which was used as a control. All data are from three technical repeats (*n* = 3). Error bars denote |S|D.

To ascertain the expression levels of *EjGIF1* in different developmental stages of loquat leaf, we then measured the expression levels of *EjGIF1* in three developmental stages of different ploidy loquats by qRT-PCR. Our results showed that, for most of the genotypes, the expression levels of *EjGIF1* displayed a tendency of rising first and then dropping, and expressed the highest levels in P II (Figure 4A).

Our previous studies on the morphologies of loquat leaves demonstrated that many morphological characteristics of triploid loquat leaves showed a different degree of heterosis compared with their parents, such as leaf length and width (Supplementary Material: Supplementary Table S1). In order to investigate the regulatory effects of *EjGIF1* on the formation of triploid leaf morphology heterosis, the expression analyses of *EjGIF1* in different ploidy loquats were performed. Based on the results above, materials of P II were used for further analysis. The results showed that the expression of *EjGIF1* in most of the hybrids exhibited AHP (A-3, A-4, A-5, A-6, A-7, A-8, and B-2) expression pattern, demonstrating pronounced heterosis (Figure 4B; Table 3). Only A-1 showed an LPL expression pattern, and A-2, A-9, B-1, and B-3 were expressed BLP (Figure 4B; Table 3). No hybrids expressed MPL and HPL. The qRT-PCR results were basically consistent with our previous morphology (leaf length and width) studies. These results indicated that *EjGIF1* may play an important role in the formation of leaf heterosis of triploid loquat.

**Table 3:** *EjGIF1* expression patterns in the hybrids.

	Additive	Dominance		Over-dominance	
	MPL^a^	HPL^b^	LPL^c^	AHP^d^	BLP^e^
*EjGIF1*	NONE	NONE	A-1	A-3, A-4, A-5, A-6, A-7, A-8, B-2	A-2, A-9, B-1, B-3

a Gene expression level was at the MPV.

b Gene expression level was at the high parent level.

c Gene expression level was at the low parent level.

d Gene expression level is above the high parent level.

e Gene expression level is below the low parent level.

### Isolation and Characterization of *EjGIF1* Promoter

**Figure 5:**
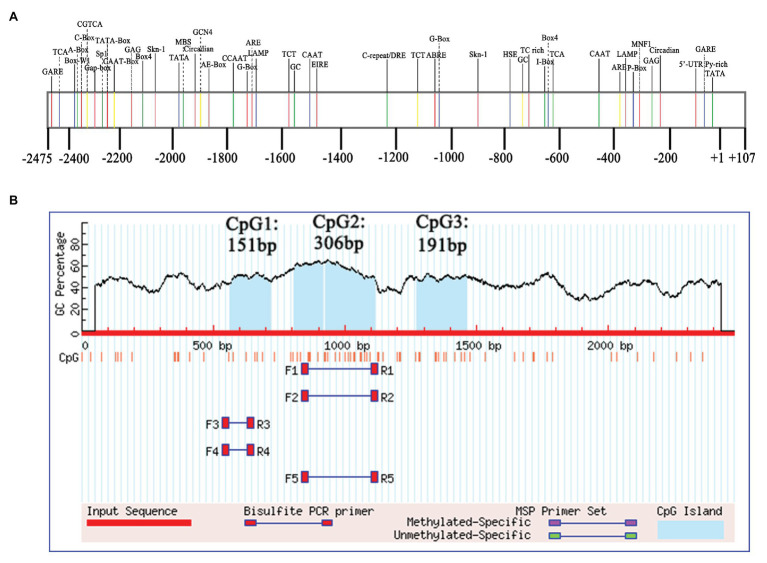
**(A)** Structure analysis and **(B)** CpG islands prediction of *EjGIF1* promoter.

Gene expression was regulated by both *Cis*-elements and *trans*-regulatory factors (Shi et al., 2012; Wittkopp and Kalay, 2012). In order to ascertain the possible regulatory mechanisms of *EjGIF1* gene in regulating the leaf development of loquats, we cloned a 2,475 bp promoter sequence from the upstream of the initiation codon of *EjGIF1* by using the Longquan-1 tetraploid genomic DNA. Results of the online prediction showed that there were five hormone-responsive elements (GARE-motif, TATC-box, TCA-element, ABRE, and P-box), 12 light-responsive elements (AE-box, Box4, C-box, G-box, GAG-motif, Gap-box, LAMP-element, Sp1, TCT-motif, CATT-motif, I-box, and MNF1), and six stress-responsive elements (HSE, ARE, GC-motif, MBS, DRE, and TC-rich repeats; Table 4; Figure 5A). What caught our attention was that the light-responsive elements were far more than the other elements, and these suggested that the expression of *EjGIF1* may be highly sensitive to light changes, but this need to be further validated.

**Table 4:** Partial *Cis*-regulatory elements in the promoter of *EjGIF1*.

	Motif	Sequence	Function
*EjGIF1*	AE-box	AGAAACAA	Part of a module for light response
	ARE	TGGTTT	*Cis*-acting regulatory element essential for the anaerobic induction
	Box 4	ATTAAT	Part of a conserved DNA module involved in light responsiveness
	C-box	CTGACGTCAG	*Cis-*acting regulatory element involved in light responsiveness
	G-box	CACGAC	*Cis-*acting regulatory element involved in light responsiveness
	GAG-motif	AGAGAGT	Part of light responsive element
	GARE-motif	AAACAGA	Gibberellin-responsive element
	Gap-box	AAATGGAGA	Part of light responsive element
	LAMP-element	CCAAAACCA	Part of light responsive element
	MBS	CAACTG	MYB binding site involved in drought-inducibility
	Sp1	CC(G/A)CCC	Light responsive element
	TATC-box	TATCCCA	*Cis*-acting element involved in gibberellin-responsiveness
	TCA-element	CCATCTTTTT	*Cis-*acting element involved in salicylic acid responsiveness
	TCT-motif	TCTTAC	Part of light responsive element
	Circadian	CAANNNNATC	*Cis-*acting regulatory element involved in circadian control
	ABRE	TACGTG	*Cis-*acting element involved in the abscisic acid responsiveness
	C-repeat/DRE	TGGCCGAC	Regulatory element involved in cold- and dehydration responsiveness
	CATT-motif	GCATTC	Part of a light responsive element
	GC-motif	CCCCCG	Enhancer-like element involved in anoxic specific inducibility
	HSE	AAAAAATTTC	*Cis-*acting element involved in heat stress responsiveness
	I-box	GATATGG	Part of light responsive element
	MNF1	GTGCCC(A/T)	Light responsive element
	P-box	CCTTTTG	Gibberellin-responsive element
	TC-rich repeats	ATTTTCTTCA	*Cis*-acting element involved in defense and stress responsiveness

### Promoter Methylation Level Analysis of Different Ploidy Loquat

**Figure 6:**
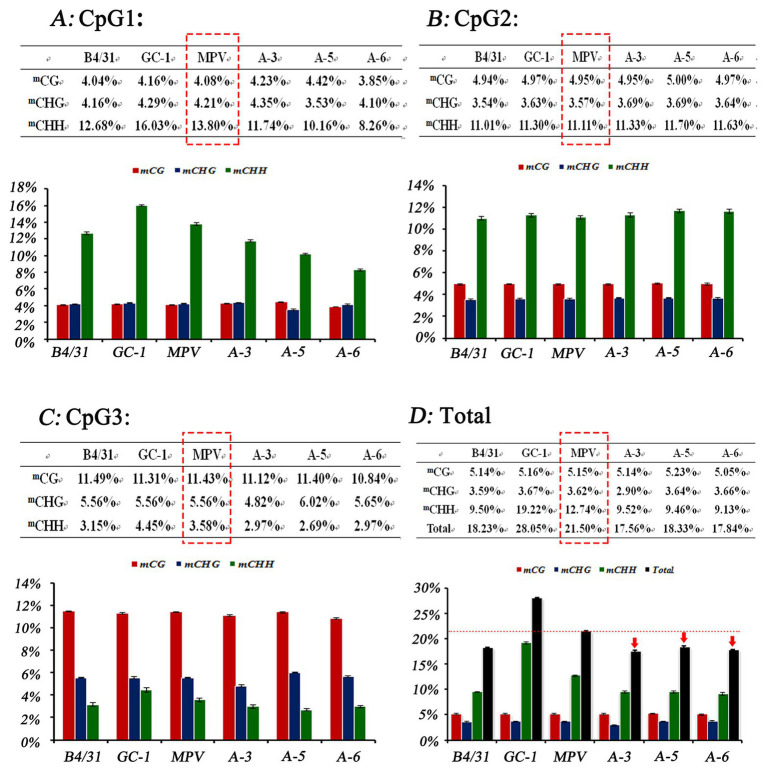
Cytosine methylation level analysis of the three CpG islands using bisulfite sequencing. Each CpG islands was sequenced by using 15 PCR clones. The collective methylation levels (%) of the three types of cytosine residues, ^m^CG, ^m^CHG, and ^m^CHH for the three CpG islands (CpG1, CpG2, and CpG3) were depicted in **(A–C)**, and the total methylation level of the promoter was depicted in **(D)**. Error bars denote |S|D.

DNA methylation level of a promoter can directly affect the transcriptional activity of the gene (Wei et al., 2018). Moreover, gene expression level could further affect the phenotype of a plant. Therefore, in order to further analyze the regulatory effects of *EjGIF1* on the leaf morphology development of loquat, we randomly selected three triploids from Triploid-A (A-3, A-5, and A-6), and the methylation levels of *EjGIF1* promoters in the three triploids and their parents (Longquan-1 tetraploid, GC-1) were analyzed by bisulfite sequencing. CpG island prediction showed that there were three CpG islands in the promoter, and the length were 151 bp, 306 bp, and 191 bp, respectively (Figure 5B). The sequences of the three CpG islands were further used for primer design (Table 1). Bisulfite sequencing results exhibited that methylation levels of diploid parent GC-1 were basically slightly higher than that of the tetraploid parent in all the three contexts (^m^CG, ^m^CHG, and ^m^CHH) among the 3 CpG islands. However, when compared with MPVs, the methylation levels of the hybrids (A-3, A-5, and A-6) showed a decreasing trend in almost all the three methylation types among CpG1 and CpG3 islands, and only CpG2 showed an increasing trend (Figures 6A–C). Interestingly, when we counted for the total methylation level for the *EjGIF1* promoter in the three hybrids, it was showed that the methylation level demonstrated a decreasing level in all the three hybrids compared with MPV (21.50%), with the methylation level of 17.56% (A-3), 18.33% (A-5), and 17.84% (A-6), respectively (Figure 6D).

Taken together, our results suggested that the total methylation levels of *EjGIF1* promoter in triploid loquats (A-3, A-5, and A-6) showed a decreasing trend, and this may generate the expression differences of *EjGIF1* between triploid loquats and parents (Longquan-1 tetraploid, GC-1), and further regulate the leaf morphology heterosis of triploid loquat.

## Discussion

Leaf is an important organ of plant photosynthesis, and it can directly affect the accumulation of sugar. In the meantime, it is also an important aspect for the plant morphology formation, and determines the growth potential of plant (Yan et al., 2008). Leaf size or leaf area greatly determines the light interception and transpiration (Monteith, 1977). Researches on leaf development have been lasted for many years. In previous studies, many transcription factors, such as GRFs or AINTEGUMENTA 3 (GIF1), that regulate leaf development have been verified and reported, and also some regulatory mechanisms of these transcription factors have been validated (Gonzalez et al., 2012; Dkhar and Pareek, 2014). Kuijt et al. (2014) found that *Oskn2*, an upstream sequence of *KNOX* gene, could interact with *OsGRF3* and *OsGRF10* in rice. In *Arabidopsis*, it was found that the expression levels of *GRFs* were regulated by *miR396*, and overexpressing *miR396* could cause narrow-leaf phenotypes (Liu et al., 2009). So far, studies on leaf development are mainly focused on the model plants, grasses, or herbaceous plants, such as *Arabidopsis*, barley, *Brassica napus* etc., and there are relatively few studies on the leaf development of woody plants (Mizukami and Fischer, 2000; Osnato et al., 2010; Liu et al., 2012; Dkhar and Pareek, 2014). In this study, we have successfully cloned a transcriptional coactivator GIF1 from loquat (EjGIF1), and our phylogenetic tree analysis showed that EjGIF1 is highly homologous with plants of the Rosaceae family, and is kept highly conserved during the evolution processes. Results of *EjGIF1* function validation demonstrated that the ectopic expression of *EjGIF1* in *Arabidopsis* could increase the leaf size, and this was consistent with previous findings (Kim et al., 2002; Horiguchi et al., 2005). Interestingly, we also found that the transgenic plantlets contained more leaves than the WTs. These results suggested that *EjGIF1* may play an important role in the leaf development of the *Arabidopsis*.

Polyploid possesses more than two sets of chromosome per cell, and it plays an important role in the plant evolution (Sattler et al., 2016). Delighting, polyploidization is often accompanied with the increased growth vigor of the plants compared with the diploid progenitors, and so does the triploid loquat (Stebbins, 1971; Chen, 2007; Li et al., 2017). Despite the ploidy effect, triploid loquat demonstrated pronounced heterosis compared with the diploid and tetraploid loquats based on our previous studies on the cultivated triploid loquats (Liu et al., 2018a,b, 2019). For the mechanisms studies of triploid loquat heterosis, some results have been obtained, but the molecular mechanisms of triploid loquat heterosis are still poorly understood (Liu et al., 2018a,b, 2019). As described above, to date, researches on the correlation between heterosis and genes are mainly on the whole genome-wide expression levels, and few studies have been performed on some specific genes. In this study, we have investigated the expression level of one specific leaf development-related gene *EjGIF1* in triploid loquats and their parents based on the results of our previous research that the leaf morphologies (length and width) of triploid loquats exhibited pronounced heterosis. Based on the results of *EjGIF1* ectopic expression in *Arabidopsis*, we further investigated the expression level of *EjGIF1* in triploid loquats and their parents. It was found that *EjGIF1* was expressed AHP in most of the triploid loquats, showing a non-additive expression pattern, and this was basically consistent with our previous studies on leaf morphology heterosis of triploid loquats (Liu et al., 2018b). These suggested that high expression of *EjGIF1* in triploid hybrids played a critical role in the leaf size heterosis formation.

Gene expression was greatly regulated by *Cis*-element, which could affect the transcriptional efficiency and stability (Garí et al., 1997; Mei et al., 2008). In order to ascertain the structure of *EjGIF1* promoter, we have successfully obtained a 2,475 bp promoter sequence by using the method of genome walking. After making a prediction for the promoter online, it was found that the light-responsive elements were significantly more than other elements, suggesting that the expression of *EjGIF1* may be greatly sensitive to light changes. In fact, many studies have found that light can affect the leaf size development, for example, light quality affects the trophic effects through photosynthesis and further determines the leaf morphogenesis or leaf area (Tardieu et al., 1999; Cookson and Granier, 2006). In this study, we indeed found that there were more light-responsive elements in the *EjGIF1* promoter, so we suggested that the expression of *EjGIF1* may be largely regulated by light changes. On the other hand, Baldissera et al. (2014) studied the alfalfa plants and found that plant branch development and the number of shoot per plant were most affected by light. Furthermore, Horiguchi et al. (2005) found that *AN3* was expressed at a high level in the basal region of leaf primordia, therefore, based on the results discussed above, we further proposed that *EjGIF1* could also promote the formation of leaf primordium. If this is the case, the transgenic *Arabidopsis* of *EjGIF1* should have more leaves than the WTs. Intriguingly, the transgenic plantlets did have more leaves than the WTs. Taken together, we speculated that the expression of *EjGIF1* was greatly induced by the light changes, and *EjGIF1* may also have an effect on the formation of leaf primordia. However, whether or how the light works on these issues are important questions and still need to be deeply studied.

Recent studies found that polyploidization could trigger extensive DNA methylation remodeling in the first or the following few generations due to the fact that it is an effective way for polyploid to maintain the genome stability (Wang et al., 2004; Fort et al., 2016). That is, DNA methylation occurred in the whole genome could regulate gene expression, inhibit transposable elements (TEs) transposition, and maintain the structure stability of chromatin (Feinberg, 2007; Bucher et al., 2012). Among them, methylation through promoter region is an effective way to inhibit gene expression without DNA sequence variation (Maunakea et al., 2010). These make more and more researchers believe that there must be a correlation between heterosis and DNA methylation, and begin to explain the heterosis mechanisms from the aspect of epigenetic (Groszmann et al., 2013; Xiong et al., 2013; Wang et al., 2018). To ascertain the methylation level of *EjGIF1* promoter among triploid loquats and their parents, we have analyzed the methylation levels in three randomly selected triploid loquats and their parents by bisulfite sequencing. It was found that the total methylation level of *EjGIF1* promoter in triploid loquats showed a decreasing trend compared with MPV, and this was consistent with the qRT-PCR results. Since the three hybrids (A-3, A-5, and A-6) used for methylation level analysis were selected randomly in this study, it was worth noting that the expression levels of *EjGIF1* in some hybrids exhibited a low expression level, and we still did not know the methylation levels of *EjGIF1* promoter in these hybrids. Therefore, the methylation levels of these low expressed hybrids need to be further detected for verifying the association between the expression level and the methylation level.

Taken together, our results suggested that (1) compared with previous studies, our study found that *EjGIF1* showed significant regulation effects on the development of leaf size; and (2) demethylation of *EjGIF1* promoter made *EjGIF1* exhibit over-dominance expression pattern in triploid loquats, and this further promoted the formation of triploid loquat heterosis. In short, *EjGIF1* played an important role in the formation of triploid loquat leaf size heterosis.

## Data Availability Statement

The datasets presented in this study can be found in online repositories. The names of the repository/repositories and accession number(s) can be found in the article/Supplementary Material.

## Author Contributions

CL and RH conducted the experiments and the paper writing. LW helped to revise the English. GL provided the funding support. All authors contributed to the article and approved the submitted version.

### Conflict of Interest

The authors declare that the research was conducted in the absence of any commercial or financial relationships that could be construed as a potential conflict of interest.
