# Supplementary material for: Functional Identification of EjGIF1 in Arabidopsis and Preliminary Analysis of Its Regulatory Mechanisms in the Formation of Triploid Loquat Leaf Heterosis
Source: Front Plant Sci. 2021 Jan 12;11:612055. doi: 10.3389/fpls.2020.612055 (PMC7835675; doi:10.3389/fpls.2020.612055)
Supplement: Supplementary file 1 [file Table_1.docx]

Table S1 leaf length and width analysis of the different ploidy loquats (Liu et al., 2018b)

| **Categories** | **Length of Leaf (LL)(cm)** | **Width of Leaf (WL)(cm)** |
| --- | --- | --- |
| Longquan-1 tetraploid | 34.12±0.7**^a^** | 13.16±0.6 |
| GC-1 | 41.28±2.0 | 15.05±0.9 |
| GC-23 | 39.20±1.4 | 14.22±1.0 |
| A-1 (LPL) | (44.3±1.6)  21.35%**^b^** | (15.18±1.0)  10.12% |
| A-2 (BLP) | (43.6±1.6)  19.54% | (15.08±1.0)  9.43% |
| A-3 (AHP) | (34.1±1.9)  -6.43% | (14.60±2.7)  5.95% |
| A-4 (AHP) | (38.58±4.2)  5.68% | (13.10±0.6)  -4.93% |
| A-5 (AHP) | (47.46±4.1)  30.00% | (17.14±1.0)  24.38% |
| A-6 (AHP) | (44.77±0.7)  22.63% | (15.28±1.2)  10.89% |
| A-7 (AHP) | (45.44±2.3)  24.47% | (15.98±0.4)  15.93% |
| A-8 (AHP) | (43.25±3.0)  18.47% | (10.68±0.5)  -22.50% |
| A-9 (BLP) | (39.98±2.0)  9.51% | (13.43±0.2)  -2.52% |
| B-1 (BLP) | (37.37±3.1)  4.34% | (13.83±0.2)  2.37% |
| B-2 (AHP) | (41.67±0.6)  16.34% | (13.80±0.4)  2.12% |
| B-3 (BLP) | (30.73±1.6)  -14.18% | (8.43±0.3)  -37.59% |

**^a^** mean ± [standard deviation](dict://key.0895DFE8DB67F9409DB285590D870EDD/standard%20deviation); **^b^** represents the Mid-parent heterosis (MPH), MPH=(F1-MPV)/MPV×100%
